# *trans*-Di­aqua­bis­(*N*,*N*,*N*′-tri­methyl­ethylenedi­amine)­nickel(II) dichloride

**DOI:** 10.1107/S2414314620011827

**Published:** 2020-09-08

**Authors:** John R. Miecznikowski, Jerry P. Jasinski, Nicole F. Flaherty, Emma E. Mircovich, Allison N. Smolinsky, Natalia R. Bertolotti

**Affiliations:** a Department of Chemistry & Biochemistry, Fairfield Univerity, 1073 North Benson Road, Fairfield, CT 06824, USA; b Department of Chemistry, Keene State College, 229 Main Street, Keene, NH 03435, USA; Vienna University of Technology, Austria

**Keywords:** Ligand precursor, crystal structure, substituted ethyl­enedi­amine ligand

## Abstract

In the title salt, [Ni(C_5_H_12_N_2_)_2_(H_2_O)_2_]Cl_2_, the asymmetric unit is comprised of half of the complex cation and a chloride ion with the Ni^II^ atom of the cation situated about a twofold rotation axis. The six-coordinate Ni^II^ atom of the cation is connected to four N atoms from two methyl-substituted ethyelenedi­amine ligands and two water mol­ecules in a slightly distorted octa­hedral environment. The five-membered chelate ring is in a slight envelope conformation. The crystal packing features O—H⋯Cl and N—H⋯Cl inter­molecular inter­actions with the Cl^−^ ion forming weak bifurcated hydrogen bonds with nearby water mol­ecules and N—H inter­actions, leading to a three-dimensional supra­molecular network structure.

## Abstract 2

The nickel(II) atom in the cation of the title salt has a slightly distorted octa­hedral coordination environment defined by two O and four N atoms.

## Graphical abstract

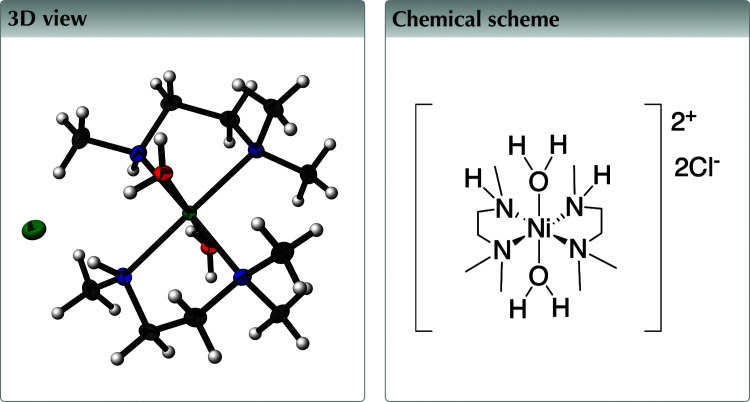

## Structure description

Previously, a tris-ethyl­enedi­amine­nickel(II) complex has been reported (Swink & Atoji, 1960 ▸). Since then, such tris-ethyl­enedi­amine complexes have been reported for nearly all of the first row transition metals as well: scandium(III) (Wagner & Melson, 1973 ▸), titanium(II) (McDonald *et al.*, 1968 ▸), vanadium(II) (Daniels *et al.*, 1995 ▸), and vanadium(III) (Clark & Greenfield, 1967 ▸), chromium(III) (Whuler *et al.*, 1975 ▸), iron(II) (Girard *et al.*, 1998 ▸), iron(III) (Renovitch & Baker, 1968 ▸), cobalt(III) (Nakatsu, 1962 ▸), copper(II) (Cullen & Lingafelter, 1970 ▸) and zinc(II) (Emsley *et al.*, 1989 ▸). Substituted tris-ethyl­enedi­amine complexes with methyl groups instead of hydrogen atoms bonded to the nitro­gen atoms have not been reported, to the best of our knowledge. In this communication, we report the preparation, spectroscopic characterization and single-crystal structure analysis of a nickel(II) complex that contains an *N,N,N*’-tri­methyl­endi­amine ligand.

**Figure 1:**
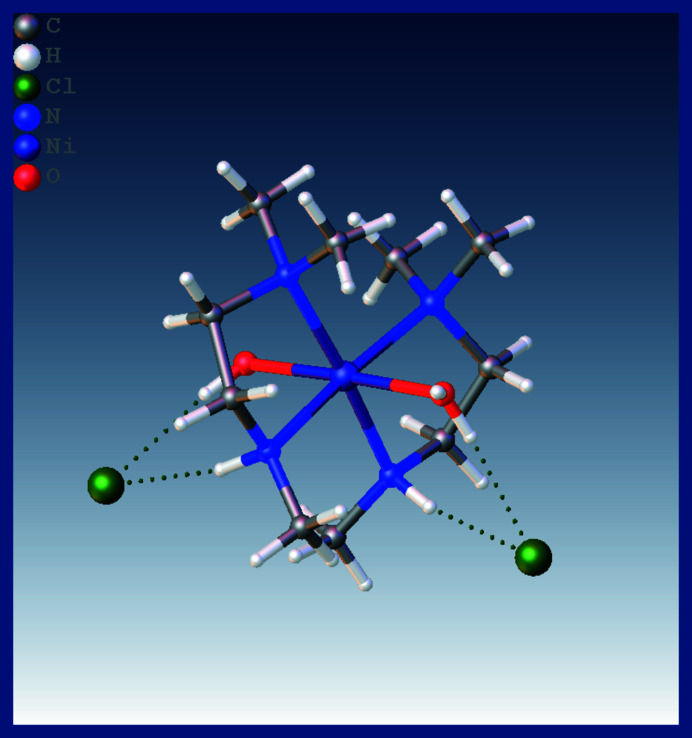
A view of [Ni(C_5_H_16_N_4_O_2_]^2+^2Cl^−^, showing its structure generated from two asymmetric units containing half of the cation complex and a chloride ion situated about a twofold rotation axis on the Ni^II^ ion. The green dotted lines represent hydrogen bonds.

**Figure 2:**
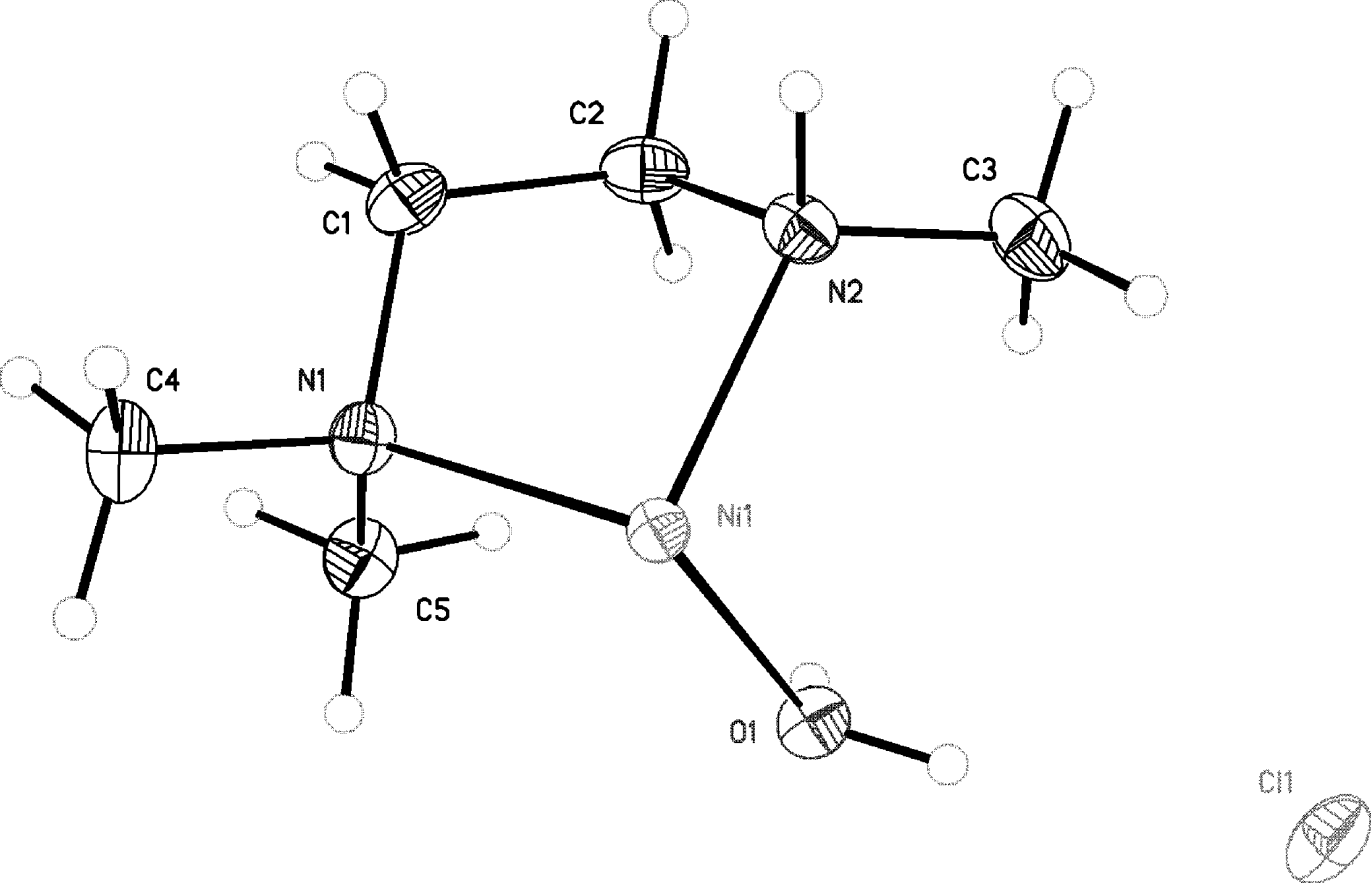
The mol­ecular structure of the asymmetric unit of [Ni(C_5_H_16_N_4_O_2_]^2+^2Cl^−^, showing the atom-labeling scheme with displacement ellipsoids drawn at the 50% probability level.

In the title salt, the asymmetric unit is comprised of half of the cationic complex and a chloride ion with the Ni^II^ atom of the cation situated about a twofold rotation axis (Fig. 1 ▸). The chelate ring (Fig. 2 ▸) is in a slight envelope conformation on C1 with puckering parameters *Q*2 = 0.476 (2)^°^ and *φ*2 = 79.8 (2)°. The six-coordinate Ni^II^ atom of the cation is connected to four N atoms from two methyl-substituted ethelenedi­amine ligands and two water mol­ecules in a slightly distorted octa­hedral environment, with the two substituted ethyl­enedi­amine ligands and two water mol­ecules each coordinating *trans* to each other. The Ni—N bond lengths of 2.1906 (18) and 2.1245 (18) Å compare well to those of 2.120 (13) Å in the literature (Swink & Atoji, 1960 ▸); the Ni—O bond of 2.1189 (15) Å is the shortest of the metal–ligand bonds.

**Figure 3:**
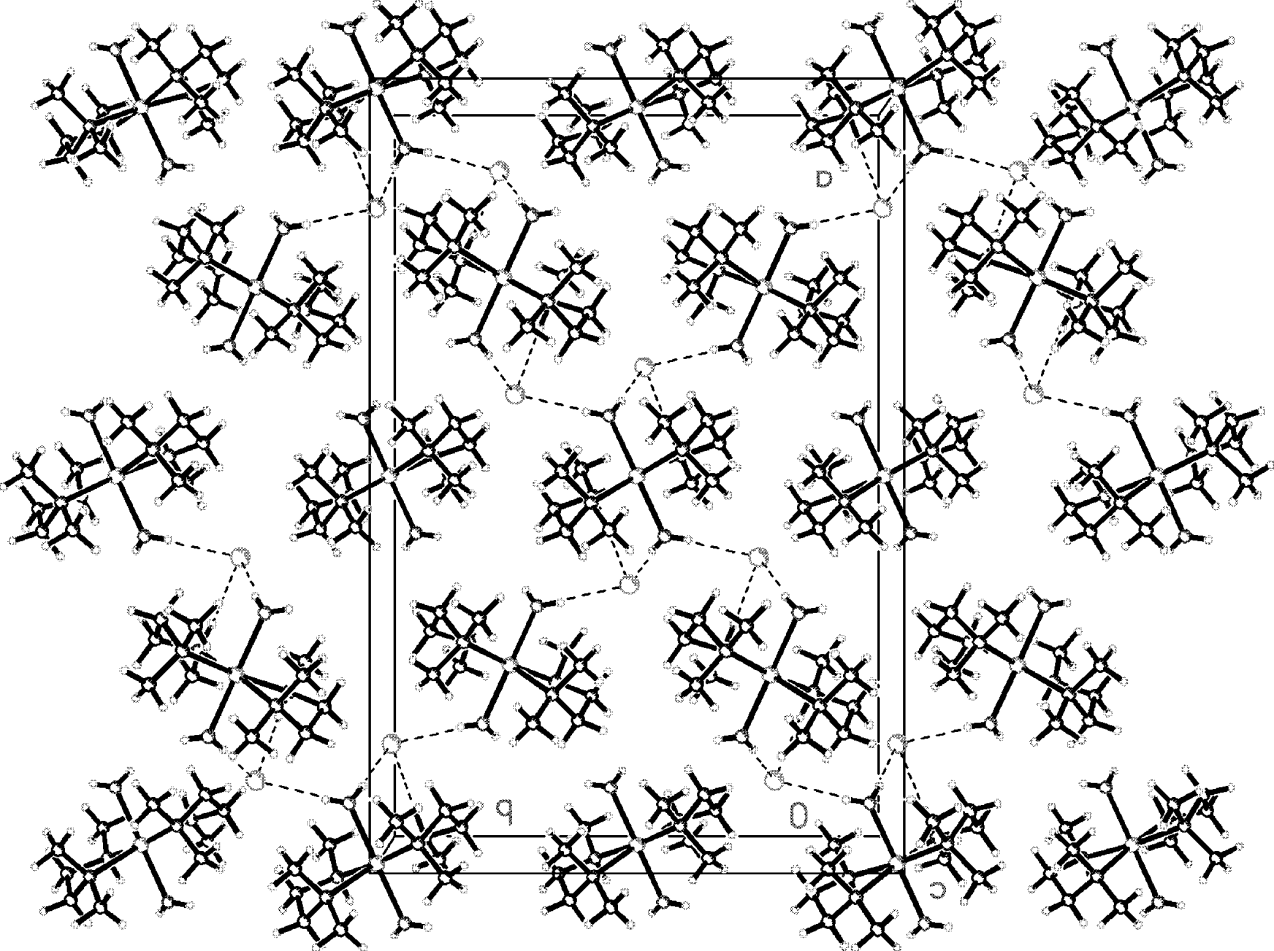
A partial packing diagram of the title compound viewed along the *c* axis showing the O—H⋯Cl and N—H⋯Cl inter­molecular inter­actions (dashed lines) with the Cl^−^ ion, forming weak bifurcated hydrogen bonds.

The crystal packing features O—H⋯Cl and N—H⋯Cl inter­molecular inter­actions with the Cl^−^ ions forming weak bifurcated hydrogen bonds with nearby water mol­ecules and N—H inter­actions from the en moieties (Fig. 3 ▸, Table 1 ▸). Chains then form along [010], [001] and [100], generating a three-dimensional supra­molecular network structure.

## Synthesis and crystallization

*N*,*N*,*N′*-Tri­methyl­ethylenedi­amine (0.47 g, 0.0046 mol) was added to 10 ml of 95%_vol_ ethanol in a round-bottom flask. To this solution, 0.32 g (0.0013 mol) of NiCl_2_·6H_2_O were added. The reaction mixture became green in color. The reaction contents were then refluxed for 18 h. After the reaction time, the solvent was removed under reduced pressure. The product was then re-dissolved in aceto­nitrile and then the aceto­nitrile was removed under reduced pressure in order to determine the yield of the product. (0.41 g, 82%). Single crystals of the product were obtained by dissolving the product in aceto­nitrile and then allowing a diethyl ether vapor to slowly diffuse into the aceto­nitrile solution which contained the product. Analysis calculated for [C_10_H_32_N_4_NiO_2_]Cl_2_: C: 32.46; H: 8.72; N: 15.14. Found: C: 32.29; H: 8.59; N: 14.96. UV–Visible data: λ (nm), (ɛ (*M* ^−1^cm^−1^) (2.4 m*M* in MeCN) 390.00 (24); 228.00 (1600); 222.00 (1700).

## Refinement

Crystal data, data collection and structure refinement details are summarized in Table 2 ▸.

## Supplementary Material

Crystal structure: contains datablock(s) I. DOI: 10.1107/S2414314620011827/wm4136sup1.cif

Structure factors: contains datablock(s) I. DOI: 10.1107/S2414314620011827/wm4136Isup2.hkl

CCDC reference: 2025810

Additional supporting information:  crystallographic information; 3D view; checkCIF report

## Figures and Tables

**Table 1:** Hydrogen-bond geometry (Å, °)

*D*—H⋯*A*	*D*—H	H⋯*A*	*D*⋯*A*	*D*—H⋯*A*
N2—H2⋯Cl1^i^	1.00	2.31	3.296 (2)	169
O1—H1*A*⋯Cl1^ii^	0.75 (3)	2.36 (3)	3.1065 (16)	172 (4)
O1—H1*B*⋯Cl1	0.85 (4)	2.24 (5)	3.0836 (19)	172 (4)

Symmetry codes: (i) 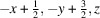 ; (ii) 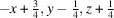 .

**Table 2:** Experimental details

Crystal data	
Chemical formula	[Ni(C_5_H_12_N_2_)_2_(H_2_O)_2_]Cl_2_
*M*_r_	370.00
Crystal system, space group	Orthorhombic, *F* *d* *d*2
Temperature (K)	173
*a*, *b*, *c* (Å)	24.7168 (8), 16.6156 (5), 8.3805 (3)
*V* (Å^3^)	3441.75 (19)
*Z*	8
Radiation type	Mo *K*α
μ (mm^−1^)	1.44
Crystal size (mm)	0.38 × 0.22 × 0.12

Data collection	
Diffractometer	Rigaku Oxford Diffraction Gemini Eos
Absorption correction	Multi-scan (*CrysAlis PRO*; Rigaku OD, 2020 ▸)
*T*_min_, *T*_max_	0.718, 1.000
No. of measured, independent and observed [*I* > 2σ(*I*)] reflections	3481, 1940, 1866
*R*_int_	0.018
(sin θ/λ)_max_ (Å^−1^)	0.758

Refinement	
*R*[*F* ^2^ > 2σ(*F* ^2^)], *wR*(*F* ^2^), *S*	0.025, 0.064, 1.03
No. of reflections	1940
No. of parameters	98
No. of restraints	1
H-atom treatment	H atoms treated by a mixture of independent and constrained refinement
Δρ_max_, Δρ_min_ (e Å^−3^)	0.45, −0.28
Absolute structure	Classical Flack method (Flack, 1983 ▸) preferred over Parsons because s.u. lower
Absolute structure parameter	0.011 (14)

Computer programs: *CrysAlis PRO* (Rigaku OD, 2020 ▸), *SHELXT* (Sheldrick, 2015*a* ▸), *SHELXL* (Sheldrick, 2015*b* ▸) and *OLEX2* (Dolomanov *et al.*, 2009 ▸).

## full crystallographic data

### Crystal data

**Table:**

[Ni(C_5_H_12_N_2_)_2_(H_2_O)_2_]Cl_2_	*D*_x_ = 1.428 Mg m^−^^3^
*M**_r_* = 370.00	Mo *K*α radiation, λ = 0.71073 Å
Orthorhombic, *F**d**d*2	Cell parameters from 1975 reflections
*a* = 24.7168 (8) Å	θ = 4.5–32.7°
*b* = 16.6156 (5) Å	µ = 1.44 mm^−^^1^
*c* = 8.3805 (3) Å	*T* = 173 K
*V* = 3441.75 (19) Å^3^	Plate, clear light blue
*Z* = 8	0.38 × 0.22 × 0.12 mm
*F*(000) = 1584	

### Data collection

**Table:**

Rigaku Oxford Diffraction Gemini Eos diffractometer	1940 independent reflections
Radiation source: fine-focus sealed X-ray tube, Enhance (Mo) X-ray Source	1866 reflections with *I* > 2σ(*I*)
Graphite monochromator	*R*_int_ = 0.018
Detector resolution: 16.0416 pixels mm^-1^	θ_max_ = 32.6°, θ_min_ = 3.7°
ω scans	*h* = −36→22
Absorption correction: multi-scan (*CrysAlisPro*; Rigaku OD, 2020)	*k* = −13→23
*T*_min_ = 0.718, *T*_max_ = 1.000	*l* = −12→5
3481 measured reflections	

### Refinement

**Table:**

Refinement on *F*^2^	Hydrogen site location: mixed
Least-squares matrix: full	H atoms treated by a mixture of independent and constrained refinement
*R*[*F*^2^ > 2σ(*F*^2^)] = 0.025	*w* = 1/[σ^2^(*F*_o_^2^) + (0.0416*P*)^2^] where *P* = (*F*_o_^2^ + 2*F*_c_^2^)/3
*wR*(*F*^2^) = 0.064	(Δ/σ)_max_ = 0.001
*S* = 1.03	Δρ_max_ = 0.45 e Å^−^^3^
1940 reflections	Δρ_min_ = −0.28 e Å^−^^3^
98 parameters	Absolute structure: Classical Flack method (Flack, 1983) preferred over Parsons because s.u. lower
1 restraint	Absolute structure parameter: 0.011 (14)
Primary atom site location: dual	

### Special details

**Table:**

Geometry. All esds (except the esd in the dihedral angle between two l.s. planes) are estimated using the full covariance matrix. The cell esds are taken into account individually in the estimation of esds in distances, angles and torsion angles; correlations between esds in cell parameters are only used when they are defined by crystal symmetry. An approximate (isotropic) treatment of cell esds is used for estimating esds involving l.s. planes.
Refinement. All of the H atoms were placed in their calculated positions and then refined with lengths of 0.99 Å (CH); 0.98 Å (CH_3_) using a riding model with *U*_iso_(H) = 1.2*U*_eq_(CH, NH) or 1.5*U*_eq_(CH_3_) of the parent atom. The idealized methyl group was refined as a rotating group. O-bound H atoms were located from a difference Fourier map and were refined freely.

### Fractional atomic coordinates and isotropic or equivalent isotropic displacement parameters (Å^2^)

**Table:**

	*x*	*y*	*z*	*U*_iso_*/*U*_eq_	
Ni1	0.250000	0.750000	0.50037 (3)	0.01242 (9)	
O1	0.32782 (6)	0.69655 (9)	0.4955 (2)	0.0176 (3)	
N1	0.21604 (8)	0.66367 (11)	0.6707 (2)	0.0168 (3)	
N2	0.22374 (8)	0.66391 (11)	0.3297 (2)	0.0178 (4)	
H2	0.190094	0.686465	0.280789	0.021*	
C1	0.17897 (9)	0.61499 (13)	0.5691 (3)	0.0220 (4)	
H1C	0.146266	0.646888	0.543559	0.026*	
H1D	0.167461	0.566293	0.628112	0.026*	
C2	0.20698 (10)	0.59057 (13)	0.4165 (3)	0.0218 (4)	
H2A	0.239070	0.557186	0.441324	0.026*	
H2B	0.182077	0.558369	0.349569	0.026*	
C3	0.25933 (11)	0.64123 (16)	0.1951 (3)	0.0259 (5)	
H3A	0.273671	0.689984	0.144676	0.039*	
H3B	0.238542	0.610325	0.116619	0.039*	
H3C	0.289369	0.608359	0.234850	0.039*	
C4	0.18262 (11)	0.69845 (14)	0.8001 (3)	0.0247 (4)	
H4A	0.206073	0.726192	0.876681	0.037*	
H4B	0.162891	0.655298	0.854570	0.037*	
H4C	0.156786	0.736879	0.754674	0.037*	
C5	0.25562 (9)	0.60896 (13)	0.7464 (3)	0.0216 (4)	
H5A	0.274396	0.577959	0.663706	0.032*	
H5B	0.236689	0.572070	0.818502	0.032*	
H5C	0.282020	0.640596	0.807014	0.032*	
Cl1	0.39455 (2)	0.76479 (3)	0.21560 (8)	0.02448 (13)	
H1A	0.3323 (12)	0.6516 (18)	0.495 (5)	0.026 (7)*	
H1B	0.3469 (19)	0.720 (2)	0.424 (5)	0.049 (11)*	

### Atomic displacement parameters (Å^2^)

**Table:**

	*U*^11^	*U*^22^	*U*^33^	*U*^12^	*U*^13^	*U*^23^
Ni1	0.01210 (15)	0.01247 (14)	0.01270 (14)	0.00142 (12)	0.000	0.000
O1	0.0163 (6)	0.0152 (6)	0.0213 (7)	0.0030 (5)	0.0004 (7)	−0.0003 (6)
N1	0.0184 (9)	0.0149 (7)	0.0172 (8)	0.0004 (6)	0.0018 (7)	0.0014 (6)
N2	0.0180 (9)	0.0181 (8)	0.0173 (8)	0.0037 (7)	−0.0023 (7)	−0.0020 (7)
C1	0.0193 (10)	0.0191 (9)	0.0274 (10)	−0.0050 (8)	0.0010 (9)	0.0010 (9)
C2	0.0223 (10)	0.0162 (8)	0.0270 (11)	−0.0012 (8)	−0.0050 (9)	−0.0036 (8)
C3	0.0315 (12)	0.0275 (11)	0.0187 (10)	0.0038 (10)	−0.0005 (10)	−0.0054 (9)
C4	0.0287 (12)	0.0233 (10)	0.0220 (9)	0.0038 (9)	0.0081 (9)	0.0024 (8)
C5	0.0237 (10)	0.0191 (9)	0.0219 (10)	0.0026 (7)	−0.0004 (10)	0.0064 (9)
Cl1	0.0205 (2)	0.0189 (2)	0.0340 (3)	−0.00289 (18)	0.0101 (2)	−0.0041 (2)

### Geometric parameters (Å, º)

**Table:**

Ni1—O1^i^	2.1189 (15)	C1—H1C	0.9900
Ni1—O1	2.1189 (15)	C1—H1D	0.9900
Ni1—N1^i^	2.1906 (18)	C1—C2	1.509 (3)
Ni1—N1	2.1906 (18)	C2—H2A	0.9900
Ni1—N2	2.1245 (18)	C2—H2B	0.9900
Ni1—N2^i^	2.1245 (18)	C3—H3A	0.9800
O1—H1A	0.75 (3)	C3—H3B	0.9800
O1—H1B	0.85 (4)	C3—H3C	0.9800
N1—C1	1.489 (3)	C4—H4A	0.9800
N1—C4	1.481 (3)	C4—H4B	0.9800
N1—C5	1.478 (3)	C4—H4C	0.9800
N2—H2	1.0000	C5—H5A	0.9800
N2—C2	1.479 (3)	C5—H5B	0.9800
N2—C3	1.479 (3)	C5—H5C	0.9800
			
O1^i^—Ni1—O1	177.80 (10)	N1—C1—H1C	109.6
O1—Ni1—N1	94.93 (7)	N1—C1—H1D	109.6
O1^i^—Ni1—N1^i^	94.93 (7)	N1—C1—C2	110.35 (18)
O1^i^—Ni1—N1	86.51 (7)	H1C—C1—H1D	108.1
O1—Ni1—N1^i^	86.51 (7)	C2—C1—H1C	109.6
O1^i^—Ni1—N2	89.54 (7)	C2—C1—H1D	109.6
O1^i^—Ni1—N2^i^	88.98 (7)	N2—C2—C1	108.91 (16)
O1—Ni1—N2^i^	89.54 (7)	N2—C2—H2A	109.9
O1—Ni1—N2	88.98 (7)	N2—C2—H2B	109.9
N1^i^—Ni1—N1	98.69 (10)	C1—C2—H2A	109.9
N2—Ni1—N1^i^	175.26 (8)	C1—C2—H2B	109.9
N2—Ni1—N1	83.15 (7)	H2A—C2—H2B	108.3
N2^i^—Ni1—N1	175.26 (8)	N2—C3—H3A	109.5
N2^i^—Ni1—N1^i^	83.15 (7)	N2—C3—H3B	109.5
N2—Ni1—N2^i^	95.36 (11)	N2—C3—H3C	109.5
Ni1—O1—H1A	123 (2)	H3A—C3—H3B	109.5
Ni1—O1—H1B	109 (3)	H3A—C3—H3C	109.5
H1A—O1—H1B	111 (4)	H3B—C3—H3C	109.5
C1—N1—Ni1	102.65 (14)	N1—C4—H4A	109.5
C4—N1—Ni1	115.81 (13)	N1—C4—H4B	109.5
C4—N1—C1	106.69 (19)	N1—C4—H4C	109.5
C5—N1—Ni1	115.38 (14)	H4A—C4—H4B	109.5
C5—N1—C1	108.56 (17)	H4A—C4—H4C	109.5
C5—N1—C4	107.15 (18)	H4B—C4—H4C	109.5
Ni1—N2—H2	106.1	N1—C5—H5A	109.5
C2—N2—Ni1	108.00 (13)	N1—C5—H5B	109.5
C2—N2—H2	106.1	N1—C5—H5C	109.5
C2—N2—C3	109.40 (18)	H5A—C5—H5B	109.5
C3—N2—Ni1	120.21 (16)	H5A—C5—H5C	109.5
C3—N2—H2	106.1	H5B—C5—H5C	109.5
			
Ni1—N1—C1—C2	46.67 (19)	C3—N2—C2—C1	170.25 (19)
Ni1—N2—C2—C1	37.8 (2)	C4—N1—C1—C2	168.89 (17)
N1—C1—C2—N2	−59.4 (2)	C5—N1—C1—C2	−75.9 (2)

Symmetry code: (i) −*x*+1/2, −*y*+3/2, *z*.

### Hydrogen-bond geometry (Å, º)

**Table:**

*D*—H···*A*	*D*—H	H···*A*	*D*···*A*	*D*—H···*A*
N2—H2···Cl1^i^	1.00	2.31	3.296 (2)	169
O1—H1*A*···Cl1^ii^	0.75 (3)	2.36 (3)	3.1065 (16)	172 (4)
O1—H1*B*···Cl1	0.85 (4)	2.24 (5)	3.0836 (19)	172 (4)

Symmetry codes: (i) −*x*+1/2, −*y*+3/2, *z*; (ii) −*x*+3/4, *y*−1/4, *z*+1/4.
